# Construction of an *n*-Body Potential for Revealing the Atomic Mechanism for Direct Alloying of Immiscible Tungsten and Copper

**DOI:** 10.3390/ma14205988

**Published:** 2021-10-12

**Authors:** Tao Zeng, Fei Li, Yuan Huang

**Affiliations:** Institute of Advanced Metallic Materials, School of Materials Science and Engineering, Tianjin University, Tianjin 300350, China; taozeng@tju.edu.cn (T.Z.); lifei14010@163.com (F.L.)

**Keywords:** W-Cu immiscible system, direct alloying, *n*-body potential, molecular dynamics simulations

## Abstract

W-Cu laminated composites are critical materials used to construct nuclear fusion reactors, and it is very important to obtain direct alloying between W and Cu at the W/Cu interfaces of the composites. Our previous experimental studies showed that it is possible to overcome the immiscibility between W and Cu and obtain direct alloying when the alloying temperature is close to the melting point of Cu. Because the W-Cu interatomic potentials published thus far cannot accurately reproduce the alloying behaviors of immiscible W and Cu, an interatomic potential suitable for the W-Cu system has been constructed in the present study. Based on this potential, direct alloying between W and Cu at high temperature has been verified, and the corresponding diffusion mechanism has been studied, through molecular dynamics (MD) simulations. The results indicate that the formation of an amorphous Cu layer at the W/Cu interface plays a critical role in alloying because it allows Cu atoms to diffuse into W. The simulation results for direct alloying between W and Cu can be verified by experimental results and transmission electron microscopy observations. This indicates that the constructed W-Cu potential can correctly model the high-temperature performance of the W-Cu system and the diffusion mechanism of direct alloying between W and Cu.

## 1. Introduction

Tungsten-copper (W-Cu) laminated composites, due to the high melting point, high sputtering resistance and low tritium retention property of W and the good thermal conductivity of Cu, have been widely used in many fields, such as plasma facing materials (PFMs), high temperature resistant materials, microelectronic materials and packaging materials [1,2,3]. However, as a typical immiscible binary metal system, the heat of formation for the W-Cu system (ΔHf = +33 kJmol^−1^) [4] is positive, which means that direct alloying between W and Cu is very difficult. Therefore, it is also difficult to prepare W-Cu laminated composites by traditional methods [5]. At present, direct alloying between W and Cu can be realized through some nonequilibrium processing techniques [6,7,8,9] that form W-Cu solid solutions. For example, some metastable W-Cu phases have been fabricated through mechanical alloying [10], ion beam mixing [11] and direct diffusion methods [12]. A typical example is the metastable hcp phase synthesized by Zhang et al. in properly designed W-Cu multilayered films through the ion beam mixing method [11]. In addition, our previous experimental studies demonstrated that it is feasible to realize direct alloying between W and Cu through high-temperature structure induced alloying (HTSIA) methods at temperatures close to the melting point of Cu (T_mCu_) [12,13], i.e., in the range from 0.81 T_mCu_ to 0.97 T_mCu_. The thermodynamic driving force for direct alloying is the surface energy and storage energy produced during the processing of raw materials [14]. This method is especially suitable for the preparation of W-Cu laminated composites.

To understand the atomic-scale structural characteristics and underlying mechanism of nonequilibrium W-Cu alloys synthesized through direct alloying, ab initio calculations and molecular dynamics (MD) simulations have been performed in recent decades [15,16,17,18]. To carry out MD simulations, some interatomic potentials [19,20] have been developed for W-Cu systems. For example, Gong et al. [19] constructed an interatomic potential and used it to carry out MD simulations to study the amorphization transition and determine the glass-forming range of the W-Cu system. Their simulation results show that W-Cu bonding occurs at low temperatures (300 K). In a study proposed by Kong et al. [20], an embedded atom method (EAM) potential was used to carry out MD simulations that showed the difficulty for Cu and W atoms to interdiffuse in the temperature range of 300–900 K. Obviously, these published W-Cu potentials are not accurate enough to explain the experimental observations of direct alloying between W and Cu by diffusion in the temperature range from 0.81 T_mCu_ to 0.97 T_mCu_. Therefore, it is necessary to construct an *n*-body potential that can be used to analyze the diffusion mechanism of direct alloying between W and Cu.

In this paper, a new *n*-body potential for the W-Cu system is constructed. The construction process roughly comprises the following steps: (a) appropriate expressions for the W-Cu potential are determined; (b) the parameters for the W-W and Cu-Cu components of the W-Cu potential are determined by solving a set of linear equations and fitting experimental values; (c) the physical properties, such as lattice constants and cohesive energies, of some fictitious solid solutions are determined by ab initio calculations and used to construct the W-Cu component of the W-Cu potential; and (d) the validity of the new W-Cu potential is verified by comparing the results of an MD simulation using the W-Cu potential with experimental results. The abovementioned MD simulation mainly applies the new *n*-body potential to simulate the physical and thermal properties of pure W and Cu and diffusion behavior during the direct alloying of W and Cu. The main experiment was to use atomic-scale resolution high-angle annular dark-field scanning transmission electron microscopy to characterize the W/Cu interface constructed through direct alloying of W and Cu. The simulation results agree well with interfacial observations and previous experimental results for alloying W and Cu, indicating that the new W-Cu potential can model W-Cu alloying and reveal the corresponding diffusion mechanism.

## 2. Methods

### 2.1. Construction of the W-Cu Interatomic Potentials

The W-Cu potentials published thus far, which are all *n*-body potentials, can be divided into two major categories: the embedded atom method (EAM) and the Finnis-Sinclair (FS) potentials [16,17]. All of them consist of three components: the W-W potential, Cu-Cu potential and W-Cu cross potential. The published W-Cu potentials are not sufficiently accurate to simulate the experimental phenomenon that is the focus of this study. We believe that the main reason for this limitation is that the mutual attraction between W atoms originating from the W-W potential part of the W-Cu potential is very strong, which eventually leads to deviations in the interaction between W and Cu in the W-Cu cross potential. One of the pieces of evidence for the strong mutual attraction between W atoms is the MD simulation of the melting point of W using these W-Cu potentials. For example, the W/Cu EAM potential presented by Gong et al. [19] was developed by modifying the *n*-body W-Cu potential constructed by Johnson et al. When this potential was used to carry out MD simulations of the melting behavior of W metal, it resulted in a very high melting point for W (4625 K) [19], while the true melting point of W is 3695 K [21]. This indicates that concerns about the melting point of W should be maintained throughout the whole construction process of the W-Cu potential. Another reason why some W-Cu potentials are not suitable to simulate the HTSIA experimental phenomenon is that the physical properties of the nonequilibrium alloy structures of the system were not fully considered during the construction process. For example, the potentials constructed by Kong et al. [20] only employed a transferable formula proposed by Johnson [21] without fitting the cohesive energies and lattice constants of the nonequilibrium alloy structures. When this W-Cu potential is used to carry out MD simulations of direct alloying between W and Cu, the simulation results predict that interdiffusion cannot occur.

To solve the above problems and construct a more accurate W-Cu potential, some methods have been adopted in this paper. The whole construction process is as follows. First, the original Finnis–Sinclair (FS) potential is adopted [22] to construct the *n*-body potential for the immiscible W-Cu system. This is mainly because the FS potential is capable of reproducing the physical properties of transition metals with bcc structures [23] based on the second-moment approximation. According to the formalisms of the original FS potential, the formula for the total $E_{tot}$ be expressed as follows [22]:

(1) $$E_{tot}=\frac{1}{2}\underset{ij}{\sum }V_{ij}\left(r_{ij}\right)+\underset{i}{\sum }F_{i}\left(\rho_{i}\right)$$

where $E_{tot}$ is the total energy of the system, $V_{ij}\left(r_{ij}\right)$ and $r_{ij}$ are the conventional central pair potential and the distance between the two corresponding atoms, respectively. $F_{i}\left(\rho_{i}\right)$ is the embedded energy, and $\rho_{i}$ is the electronic density at atom *i* due to all its neighbors.

According to Ackland et al. [24], because of the electronic structure difference, the original FS formalism is not very effective for face-centered cubic (fcc) metals, and calculations that use the FS potential to determine thermal expansion coefficients of crystals produce values that deviate from experimental values. Furthermore, extending the FS formalism from pure metals to alloys is relatively inconvenient. Accordingly, K. P. Tai [25] proposed the extended-FS potential, which provides an extension to the original FS formalism and has a simple analytic form. The extended-FS potential can correctly describe the physical properties of some noble metal systems with an fcc structure. Therefore, at the present time, the specific expressions for the central pair potentials $V_{W}\left(r\right)$ and $V_{\mathrm{Cu}}\left(r\right)$ in Equation (1) are given by the following formulas, where Equation (2) [22] is the original pair potential for W with a bcc structure and Equation (3) [26] is the extended pair potential for Cu with an fcc structure,

(2) $$V_{W}\left(r\right)=\{\begin{array}{ll} {\left(r-c\right)}^{2}\left(c_{0}+c_{1}r+c_{2}r^{2}\right), & r\leq c\\ 0, & r>c \end{array}$$

(3) $$V_{\mathrm{Cu}}\left(r\right)=\{\begin{array}{ll} {\left(r-c\right)}^{2}\left(c_{0}+c_{1}r+c_{2}r^{2}+c_{3}r^{3}+c_{4}r^{4}\right), & r\leq c\\ 0, & r>c \end{array}$$

where *r* is the interatomic distance, *c* is the cutoff distance of the pair potential, and $c_{0}$, $c_{1}$, $c_{2}$, $c_{3}$ and $c_{4}$ are the potential parameters to be fitted.

The specific expressions of the embedded energy ($F_{i}\left(\rho_{i}\right)$) and electronic density ($\rho_{i}$) in Equation (1) are as follows [22,26]:

(4) $$F\left(\rho_{i}\right)=-\sqrt{\rho_{i}}$$

(5) $$\rho_{i}=\underset{i\neq j}{\sum }A^{2}\phi_{ij}\left(r_{ij}\right)$$

(6) $$\phi_{W}\left(r\right)=\{\begin{array}{c} {\left(r-d\right)}^{2},r\leq d\\ 0,\begin{array}{ccc} & & r>d \end{array} \end{array}$$

(7) $$\phi_{\mathrm{Cu}}\left(r\right)=\{\begin{array}{ll} {\left(r-d\right)}^{2}+b^{2}{\left(r-d\right)}^{4}, & r\leq d\\ 0, & r>d \end{array}$$

where *r* is the distance between the atoms, *d* is the electronic density function, and *b* is the potential parameter.

The formalism of the extended-FS potential is used to construct the W-Cu component of the W-Cu potential. Correspondingly, the extended pair-potential ($V^{W-\mathrm{Cu}}\left(r^{W-\mathrm{Cu}}\right)$), the embedded energy ($F^{W-\mathrm{Cu}}\left(\rho_{i}^{W-\mathrm{Cu}}\right)$) and the electronic density ($\rho_{i}^{W-\mathrm{Cu}}$) can be expressed as follows.

(8) $$V^{W-\mathrm{Cu}}\left(r^{W-\mathrm{Cu}}\right)=\{\begin{array}{l} {\left(r^{W-\mathrm{Cu}}-c\right)}^{2}(c_{0}+c_{1}r^{W-\mathrm{Cu}}+c_{2}{\left(r^{W-\mathrm{Cu}}\right)}^{2}+c_{3}{\left(r^{W-\mathrm{Cu}}\right)}^{3}+c_{4}\left(r^{W-\mathrm{Cu}}{)}^{4}\right),r^{W-\mathrm{Cu}}\leq c\\ 0,r^{W-\mathrm{Cu}}>c \end{array}$$

(9) $$F^{W-\mathrm{Cu}}\left(\rho_{i}^{W-\mathrm{Cu}}\right)=-\sqrt{\rho_{i}^{W-\mathrm{Cu}}}$$

(10) $$\rho_{i}^{W-\mathrm{Cu}}=\underset{j\neq i}{\sum }A^{2}\phi^{W-\mathrm{Cu}}\left(r^{W-\mathrm{Cu}}\right)$$

(11) $$\phi^{W-\mathrm{Cu}}\left(r^{W-\mathrm{Cu}}\right)=\{\begin{array}{l} (r^{W-\mathrm{Cu}}-d{)}^{2}+b^{2}{\left(r^{W-\mathrm{Cu}}-d\right)}^{4},r^{W-\mathrm{Cu}}\leq d\\ 0,r^{W-\mathrm{Cu}}>d \end{array}$$

where $r^{W-\mathrm{Cu}}$ is the distance between the W and Cu atoms, *c* and *d* are the cutoff distances of the extended pair potential and electronic density, respectively, and $c_{0}$, $c_{1}$, $c_{2}$, $c_{3}$, $c_{4}$ and b are the potential parameters to be fitted.

According to the results of tight-binding theory [22], the *n*-body term—namely, the embedded energy $F_{i}\left(\rho_{i}\right)$ in Equation (4), is proportional to the square root of the total electron density function $\rho_{i}$ at atom *i*. The $\rho_{i}$ at atom *i* can be approximated by the linear superposition of the electron density $\phi \left(r_{ij}\right)$ contributed by the neighboring atoms, which is a reasonable simplification according to the second-moment approximation [19]. The cutoff parameters *c* and *d* are assumed to lie between the second nearest-neighbor and third nearest-neighbor atoms of each atom in the corresponding structure. For the bcc structure, the cutoff parameters are between *a* and sqrt (2)*a, and for the fcc structure, they are between a and sqrt (6)/2*a, where *a* is the lattice constant of the corresponding structure. When the parameters *c*_3_ and *c*_4_ in Equation (3) and the parameter *b* in Equation (7) are set to zero, the extended-FS potential is reduced to the original form.

Thus, in the present study, nine parameters (A, d, c, $c_{0}$, $c_{1}$, $c_{2}$, $c_{3}$, $c_{4}$, b) were fitted for the Cu-Cu potential part of the W-Cu potential, and another six parameters (A, d, c, $c_{0}$, $c_{1}$, $c_{2}$) were fitted for the W-W potential part of the W-Cu potential. In addition, some experimental physical property data, such as the cohesive energy ($E_{c}$), lattice constant (*a*), vacancy formation energies ($E_{v}$), bulk modules (B), elastic constants ($C_{11}$, $C_{12}$, and $C_{44}$), and pressure (*P*), are applied to determine the Cu-Cu and W-W potential parts of the W-Cu potential. The relationships between these physical properties and the corresponding potentials are described in Section A of the Supporting Information for this paper. The process for determining the parameters for the W-W potential part of W-Cu potential is to solve a set of linear equations by an iteration on the parameter d with the experimental values of the cohesive energy, lattice constant, bulk modulus and Cauchy pressure in this paper, which is a reasonable approximation of the method proposed by Finnis and Sinclair [22]. For the Cu-Cu potential part of the W-Cu potential, the corresponding potential parameters are obtained through a least squares method that has been applied to minimize the fitting error of Formula (12).

(12) $$ER={\left(E_{C}-E_{C}^{e}\right)}^{2}+P^{2}+{\left(E_{v}-E_{v}^{e}\right)}^{2}+{\left(C_{11}-C_{11}^{e}\right)}^{2}+{\left(C_{12}-C_{12}^{e}\right)}^{2}+{\left(C_{44}-C_{44}^{e}\right)}^{2}$$

where $E_{c}^{e}$, $E_{v}^{e}$, $C_{11}^{e}$, $C_{12}^{e}$ and $C_{44}^{e}$ represent the experimental values of the cohesive energy ($E_{c}$), vacancy formation energies ($E_{v}$) and elastic constants ($C_{11}$, $C_{12}$, and $C_{44}$), respectively. The ER term in Equation (8) represents the fitting error. A simple computer program is adopted to solve the problem of fitting optimization.

The W-Cu cross-potential part of the W-Cu potential has the same formalism as the Cu-Cu potential (see Equations (8)–(11)), and the fitting process is also similar to the Cu-Cu potential part of the W-Cu potential. There are very few experimental data available for deriving the parameters of the W–Cu cross potential since the W-Cu system is essentially immiscible. To solve this problem, considering that simple structures form more easily in immiscible systems and referring to preexisting studies [18], two fictitious W-Cu solid solutions with the selected structures of B2 and L1_2_ have been constructed. Then, by using the well-established Vienna ab initio simulation package (VASP) [27,28], ab initio calculations of the lattice constants and cohesive energies were carried out for the nonequilibrium fictitious W-Cu solid solution structures. The fictitious W-Cu solid solution structures and corresponding calculation process are described in the Supporting Information for this paper. The calculation results were eventually used to fit the parameters of the W-Cu cross-potential.

### 2.2. Simulation Details

To validate the W-W and Cu-Cu potential parts of the W-Cu potential, the new potential is used to calculate the physical properties of pure W and Cu metals, including the cohesive energies, lattice constants, vacancy formation energies, elastic constants and melting points of W and Cu, and the results are compared with corresponding experimental values. The calculated melting point can be obtained through the two-phase simulation (TPS) method, which is more accurate than the one-phase simulation (OPS) method according to Arima et al. [29] The initial supercell with coexisting solid and liquid phases contains 20 ∗ 20 ∗ 20 ∗ 2 unit cells. During the MD simulations, the initial supercell was relaxed in the isothermal-isobaric (NPT) ensemble at desired temperatures and pressures to reach the equilibrium state. Then, the melting point was determined based on the density change [15].

**Figure 1:**
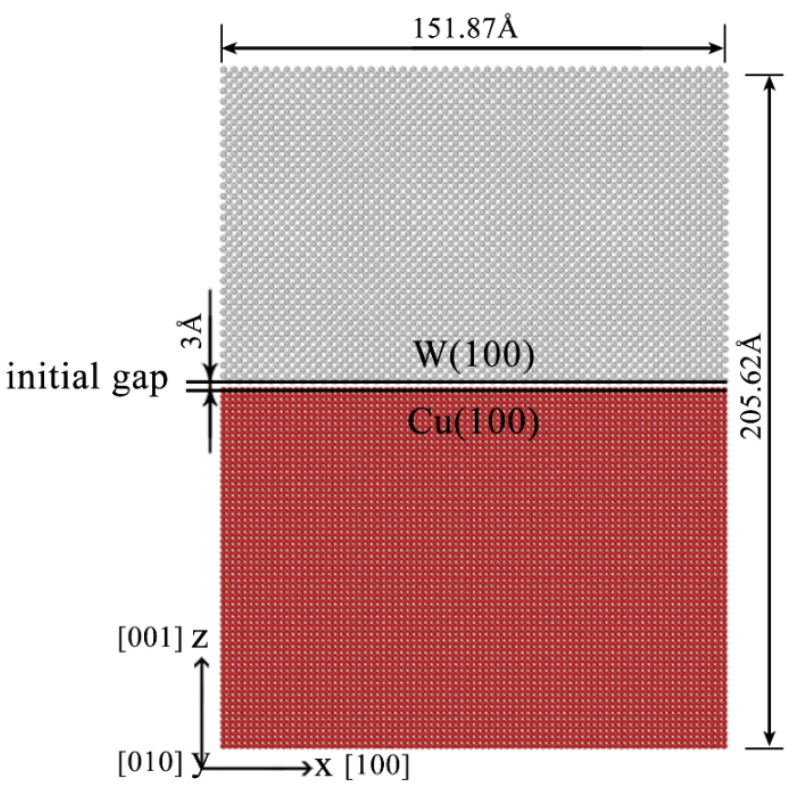
Initial interface model used for molecular dynamic simulations of direct alloying between Cu and W, where the upper layer is the W layer, and the lower layer is the Cu layer.

To understand the atomic-scale structural characteristics and underlying mechanism of nonequilibrium W-Cu alloys, a classical molecular dynamics code (LAMMPS [30]) based on the theoretical framework of Newtonian mechanics has been applied to simulate direct alloying. Through the simulation, the atomic-scale structure evolution, diffusion mechanism and influence of temperature during direct alloying can also be revealed. In the present study, as shown in Figure 1, a W-Cu interface simulation model consisting of 211,680 Cu atoms and 138,240 W atoms was constructed. The dimensions of the unit cell were 42 ∗ 42 ∗ 30 ($a_{\mathrm{Cu}}$) and 48 ∗ 48 ∗ 30 ($a_{W}$), where $a_{\mathrm{Cu}}$ and $a_{W}$ were the lattice constants of Cu and W, respectively. The reason for setting the sizes was because 48/42 = 1.143 was very close to $a_{\mathrm{Cu}}/a_{W}$ = 1.144. The contact surface between the upper (W) and lower (Cu) layers is an ideal (100) crystal plane with the [1 0 0], [0 1 0] and [0 0 1] directions parallel to the *x*, *y*, and *z* axes, respectively. To avoid strong interactions when W and Cu atoms were extremely close, a 3 Å initial gap was added between the W and Cu layers at the initial interface [31]. In the x- and y-axes of the model, periodic boundary conditions were applied. Translational motions in the *z*-axis direction of the five atomic layers in the lower surface of Cu and the upper surface of W were fixed during the simulation.

During the simulations, the whole system was first relaxed in the NPT ensemble with zero external pressure at 300 K for 100 ps. After this initial equilibration run, the system was rapidly heated to the selected simulation temperature. Considering the necessary temperatures for atoms at the interface to diffuse into the opposite side are commonly between 0.6-0.8 *T*_m_ [32] and the melting points of Cu and W are 1358 K and 3695 K, respectively, the simulation temperatures were chosen as 900 K, 1000 K, 1100 K, 1200 K and 1300 K. Subsequently, the simulation of the interface model runs at the specified temperatures in the same NPT ensemble to achieve a satisfactorily stable state in which the thermodynamic data of the system do not change significantly. The temperature of interdiffusion of W and Cu was determined by looking for the temperature at which diffusion can obviously occur according to the simulation results.

The diffusion mechanism of direct alloying between Cu and W can be discussed by analyzing the atomic coordinates, concentration distribution curves, pair correlation functions (g(r)) and fine-scale density profiles ($\rho_{\alpha }\left(z\right)$) obtained from the final simulation results. The concentration distribution curves were calculated to define the distribution of each component along the z axis, from which the diffusion distance during alloying between W and Cu can be determined. The analyses of the pair correlation functions (g(r)) and the fine-scale density profiles ($\rho_{\alpha }\left(z\right)$) are described in Sections C and D of the Supporting Information for this paper, respectively.

## 3. Results and Discussion

### 3.1. Validation of the New W-Cu Potential through the Simulation of Physical Properties

The obtained parameters of the Cu-Cu, W-W and W-Cu components of the W-Cu potential in this paper are listed in Table 1.

As stated above, the physical properties of pure W and Cu metals, including the cohesive energies, lattice constants, vacancy formation energies, elastic constants and melting points, were calculated using the MD simulation method and the new W-Cu potentials to verify the reliability of the Cu-Cu and W-W potentials. The simulation results and experimental data [33,34] for the cohesive energies, lattice constants, vacancy formation energies, and elastic constants are listed in Table 2. Table 2 shows that the derived W-Cu potential works fairly well in reproducing the physical properties of pure W and Cu.

**Figure 2:**
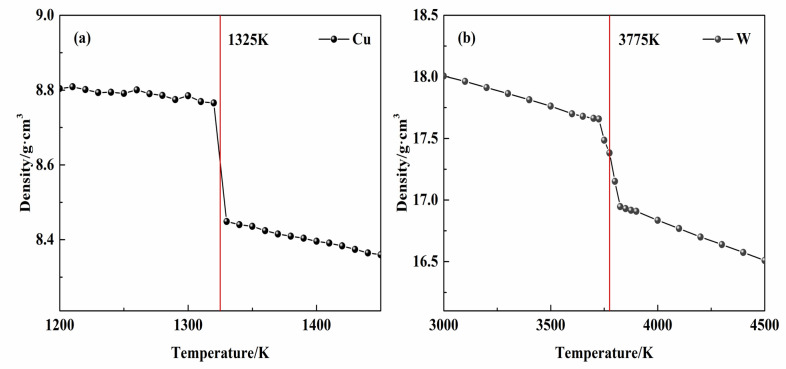
Relationship between (**a**) Cu (**b**) W density and temperature.

Figure 2 shows the densities of W and Cu obtained by the TPS method at different temperatures. Thus, the calculated melting points are 1325 K for Cu and 3775 K for W, which are relatively consistent with the experimental values (1358 K and 3695 K, respectively) [35]. The melting point of W calculated through the W-Cu potential constructed by Gong et al. [19] is approximately 4625 K, which is 930 K higher than the experimental value.

The cohesive energies and lattice constants of the three nonequilibrium phases, Cu3W, CuW and CuW3, obtained by MD simulations using the new W-Cu potential and ab initio calculations are listed in Table 3. Table 3 shows that the properties calculated from the new W-Cu potential are in good agreement with those obtained from ab initio calculations. Evidently, the new W-Cu potential work fairly well in terms of reproducing some physical properties of pure Cu and W, as well as the nonequilibrium phases in the system.

### 3.2. MD Simulation for Direct Alloying between W and Cu Via the New W-Cu Potential

As stated above, to understand the atomic-scale structural characteristics of nonequilibrium W-Cu alloys, MD simulations were carried out using the W-Cu potential. In addition, Zhang et al. [14] proposed that direct alloying between immiscible W and Cu near the melting point of Cu is thermodynamically possible. It is necessary to understand the diffusion mechanism of direct alloying between W and Cu at different temperatures close to the melting point of Cu, which is the reason we carried out MD simulations of alloying with the new W-Cu potential.

**Figure 3:**
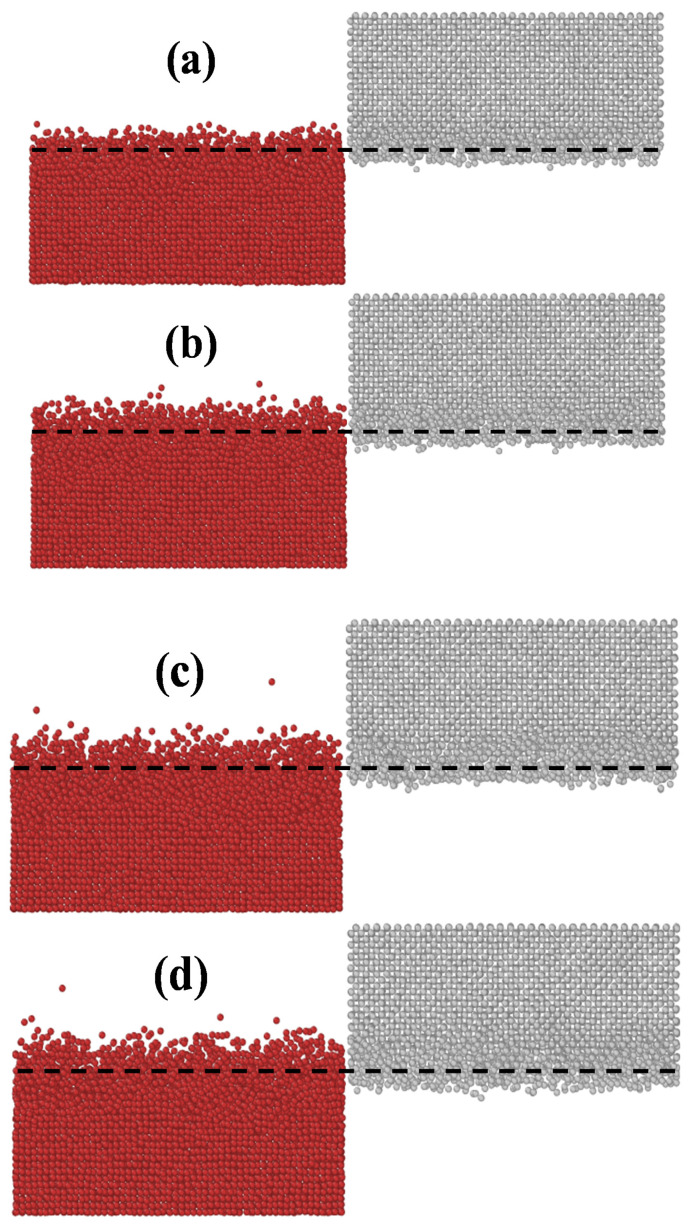
W/Cu interfacial structures obtained through alloying at a temperature of 1200 K for different times: (**a**) 0.2 ns, (**b**) 1 ns, (**c**) 2 ns, and (**d**) 4 ns. The red and gray atoms represent Cu and W atoms, respectively. These results are obtained from MD simulations using the new W-Cu potential.

Figure 3 shows the W/Cu interfacial structures obtained through MD simulations of alloying at 1200 K for different lengths of time. To analyze the distribution of atoms at the interface, the W block was shifted to the right by 152 Å. Figure 3 shows that diffusion occurred between W and Cu, and the number of atoms involved in diffusion and the diffusion distance increased with time, indicating that direct alloying between W and Cu occurred at this temperature. When the simulation time is 0.2 ns, the number of interdiffused atoms is only 0.94% of the total number of atoms, and the percentage reaches 1.54% when the simulation time is 2 ns. When the simulation time is 4 ns, the diffusion distance does not increase significantly, indicating that the increase in the diffusion distance tends to stabilize with increasing time. This observation agrees well with the observation by Chen et al. [32] for a Cu-Al system at high temperatures. Meanwhile, Figure 3 shows that the diffusion of the two kinds of atoms is not symmetric. The amount of Cu atoms diffusing into the W matrix is greater than that of W atoms involved in mutual diffusion, and the W atoms prefer to stay at the interface. This result has also been observed in experiments by Jiang et al. [36] in X-ray diffraction (XRD) tests that were carried out for the W/Cu interface constructed through direct alloying. The XRD results show that the W peaks at the interfaces shift toward larger 2θ angles, which is the result of the dissolution of Cu atoms into W. We believe that Cu diffuses into W rather than W into Cu mainly due to the atomic sizes of Cu and W. The atomic radius of W (~1.40 Å [37]) is larger than that of Cu (~1.28 Å [37]). Some studies [32,38] show that atoms with smaller radii more easily diffuse into metals whose atoms have larger radii. Thus, it is not difficult to conclude that Cu more easily diffuses into W, while W has relatively difficulty diffusing into Cu during alloying. The pairwise correlation functions *g*(*r*) of the W/Cu interfacial structure at different times are calculated according from the MD simulation results (see Section C of the Supporting Information), which characterize the W/Cu alloying structure after alloying.

**Figure 4:**
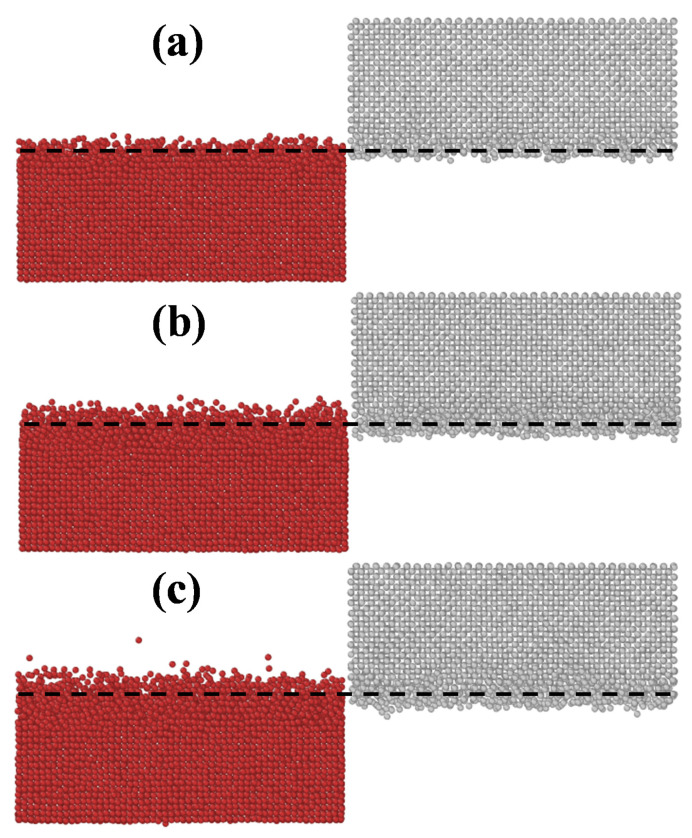

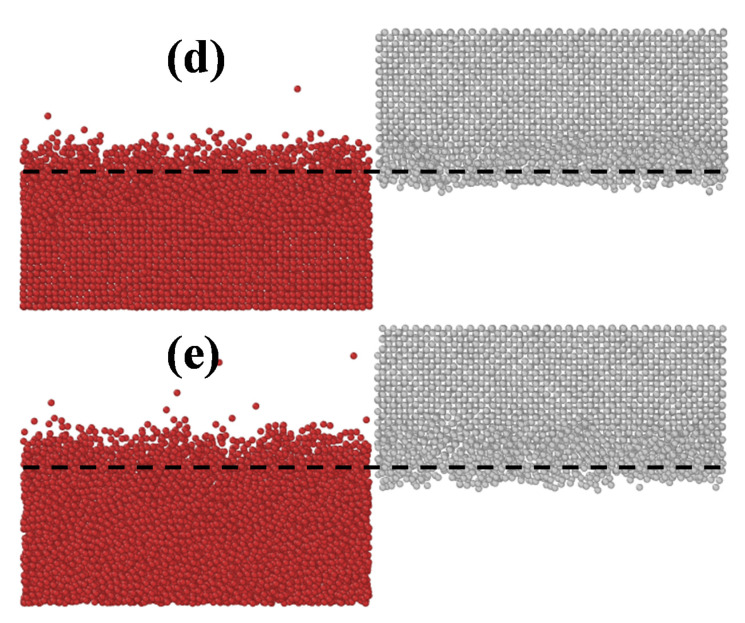
The W-Cu interfacial structures after simulation for 2 ns at different temperatures: (**a**) 900 K, (**b**) 1000 K, (**c**) 1100 K, (**d**) 1200 K, and (**e**) 1300 K. The red and gray atoms represent Cu and W atoms, respectively.

**Figure 5:**
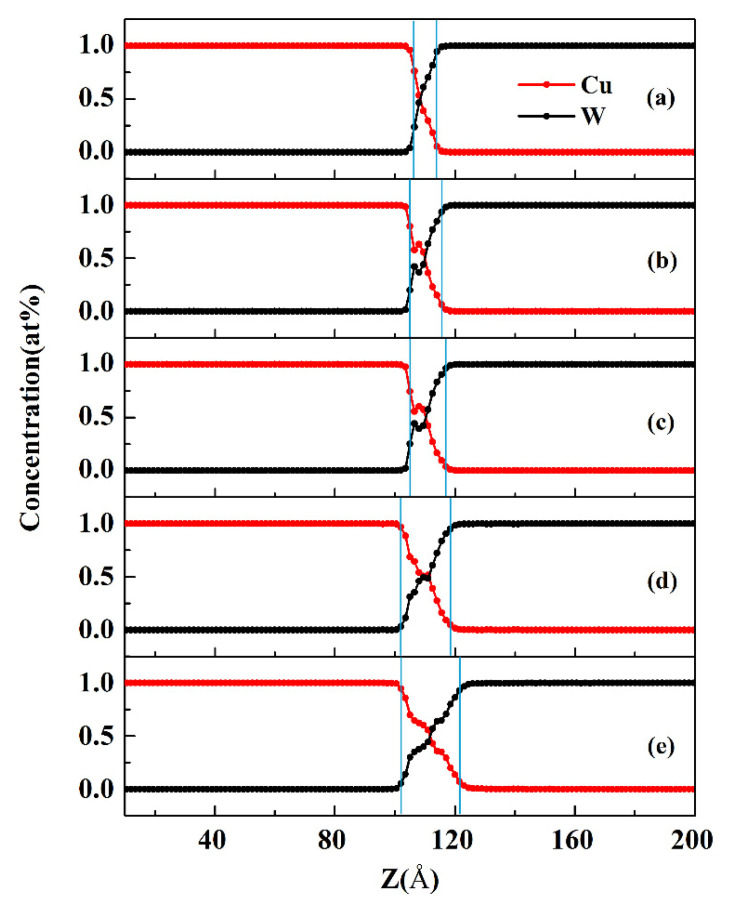
The W-Cu diffusion curves after simulation for 2 ns at different temperatures: (**a**) 900 K, (**b**) 1000 K, (**c**) 1100 K, (**d**) 1200 K, and (**e**) 1300 K.

MD simulations of the W-Cu direct alloying process using the new W-Cu potential were carried out in this paper. During the simulations, the alloying temperatures were set at 900 K, 1000 K, 1100 K, 1200 K and 1300 K, and the alloying time was set at 2 ns. The W/Cu interfacial structure and the diffusion curves are shown in Figure 4 and Figure 5, respectively. The regions where the concentration of solute atoms is greater than 5% are defined as the interface layers [32]. Figure 4 shows that when the alloying temperature is 900 K (see Figure 4a), the diffusion distance is approximately 4.5 Å (see Figure 5a), indicating very little diffusion across the interface. When the alloying temperature is 1000 K (see Figure 4b), more Cu atoms begin to diffuse into W, and the structure of Cu begins to become disordered at the interface. With increasing temperature, the diffusion of Cu atoms becomes obvious. When the alloying temperature reaches 1200 K (see Figure 4d), the number of Cu atoms diffusing into the W region increases, causing the original ordered structure of W to gradually become disordered. At this time, as shown in Figure 5d, the thickness of the diffusion layer obtained is approximately 13.5 Å. When the temperature reaches 1300 K (see Figure 4e), the diffusion increases significantly, and the diffusion distance reaches 16.5 Å (see Figure 5e).

Obviously, the above simulation results show that the diffusion distance increases rapidly with increasing temperature, and there is a temperature range from 900 K to less than T_mCu_ for alloying between W and Cu, which is consistent with the experimental results provided by Zhang et al. [14], that the bonding temperatures for W and Cu should be controlled in an effective temperature range 0.81 T_mCu_-0.97 T_mCu._ This result indicates that the new W-Cu potential can reasonably describe the diffusion process in a W-Cu system at high temperature. In contrast, when the EAM W-Cu potential presented by Zhou et al. [39] is used to carry out a MD simulation of direct alloying of W and Cu, diffusion is not observed. For another example, when the W-Cu potential constructed by Gong et al. [19] is used to carry out MD simulations of the direct alloying of W and Cu, diffusion can occur at room temperature, which is unreasonable.

**Figure 6:**
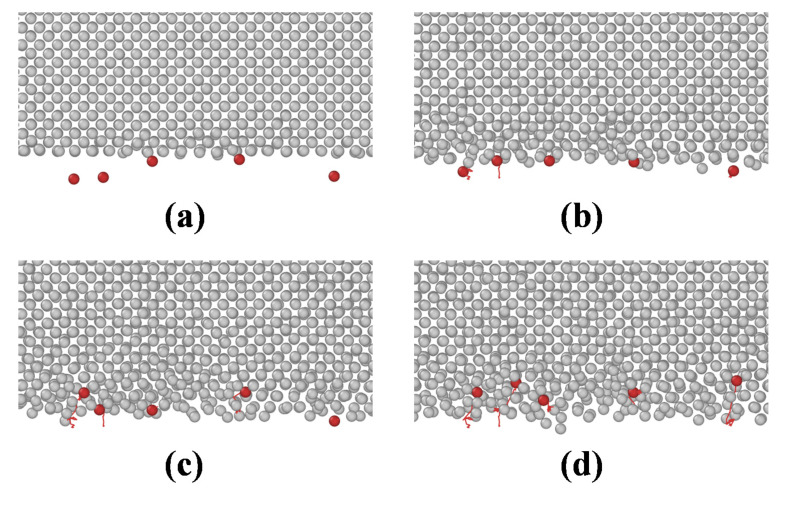
Particle trajectories of labeled Cu atoms at different alloying temperatures: (**a**) 300 K, (**b**) 900 K, (**c**) 1100 K, and (**d**) 1300 K.

To better understand the mechanism of direct alloying between W and Cu, some Cu atoms were labeled using Ovito software. According to the MD simulation results, the particle trajectories of these labeled Cu atoms were analyzed and are shown in Figure 6. Figure 6 shows that Cu atoms gradually squeeze into W with increasing alloying temperature. Meanwhile, the squeezing process leads to structural disorder of W, which further promotes the squeezing of Cu atoms.

The results of the fine-scale density profiles (see Section D of the Supporting Information) also indicate that with increasing alloying temperature, Cu becomes gradually disordered at and near the interface, while W only becomes disordered at the interface. These analyses support the previous conclusion that during alloying, the structure of Cu becomes disordered, and the structure of W becomes relatively relaxed, which weakens the binding strength and motion resistance of Cu atoms and realizes the diffusion of Cu atoms into W metal.

### 3.3. Experimental Verification of the New W-Cu Potential

The results of MD simulation show that there are three main characteristics of W-Cu alloying: (1) the temperature range for alloying; (2) mainly Cu atoms diffuse into W metal during the alloying process; and (3) although the alloying temperature does not reach the melting point of Cu, a disordered amorphous structure develops in the Cu metal, which makes the Cu atoms break free from the binding. The first and second points have been verified by previous experimental studies [12,36]. To further verify the reliability of the new W-Cu potential, it will be necessary to observe the interface after W-Cu alloying to confirm the existence of amorphous Cu. Thus, we performed direct alloying experiments of diffusion bonding using W and Cu rods, which was the same method used in previous work [12]. In the experiment, the contact surfaces of the W and Cu rods were first polished to a mirror finish. Then, high-precision coaxial assembly of the W and Cu rods was carried out using a fixture. Finally, the W-Cu assembly was annealed in a hydrogen atmosphere at 980°C for 180 min with an external axial pressure of 106 MPa. According to our previous studies [14], direct alloying between W and Cu can be realized in the range 0.81 T_mCu_~0.97 T_mCu_. Therefore, when annealing was completed, alloying between W and Cu was also completed, and there should be a metallurgical bonding interface directly between W and Cu. Therefore, the characterization of the W/Cu interface in the W/Cu joint can be used to verify the MD simulation results of direct alloying between W and Cu and then verify the new W-Cu potential. Characterization of the W-Cu interface was carried out with atomic-scale resolution high-angle annular dark-field scanning transmission electron microscopy (HAADF-STEM, JEM-ARM200F). Before interfacial characterization, the W-Cu TEM specimens were prepared by cutting the W/Cu joint and thinning with the focused ion beam (FIB) technique.

**Figure 7:**
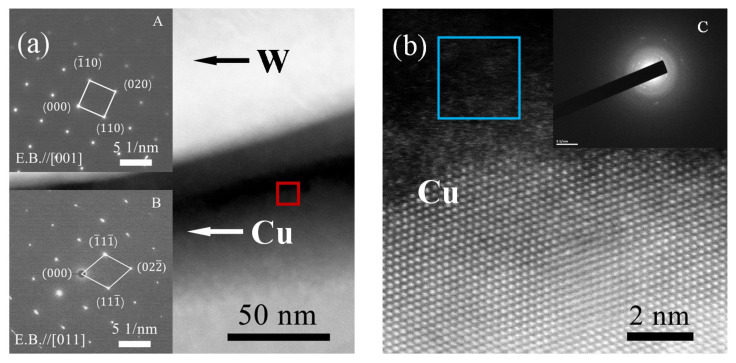
(**a**) HAADF-STEM image of the W/Cu interface through direct alloying at 980 °C; (**b**) Atomic resolution HAADF-STEM images of the region marked with a red rectangle in (**a**).

The characterization results are shown in Figure 7, where Figure 7a is a HAADF-STEM image of the W/Cu interface. The insets of Figure 7a are the corresponding selected areas of electron diffraction (SAED) patterns of the bright and dark areas in Figure 7a. It should be pointed out that the SAED patterns of the bright and dark areas were obtained under different orientations, since there exist deviations in the crystal orientation on both sides of the interface. Inset (A) exhibits a bcc crystal structure along the [001] direction and reveals that the bright area is W. Inset (B) exhibits an fcc crystal structure along the [011] direction and reveals that the dark area is Cu. Figure 7b shows the atomic resolution HAADF-STEM images of the region marked with a red rectangle in Figure 7a. The SAED pattern of the region marked with a blue rectangle (Inset (C)) shows that there exists an amorphous region, mainly composed of Cu, at the interface. Because the alloying temperature of 980 °C does not reach the melting point of Cu, the amorphous region of Cu should form during alloying rather than melting, which is evidence for the formation of the disordered structure of Cu, as shown by the MD simulation. The third characteristic of W-Cu alloying concluded from the MD simulation results is also confirmed by experimental results, indicating that the new W-Cu potential in this study can accurately model the alloying behavior of immiscible W-Cu systems.

### 3.4. Fundamental Reason for the Ability of the New Potential to Model the Alloying Behavior of the W-Cu System

We believe that the fundamental reason that the new W-Cu potential in this paper can more accurately model the alloying behavior of W and Cu at high temperature is that the melting points of the two elements, especially W, have been accurately modeled. The melting point of a pure metal characterizes the mutual attraction between the atoms of the pure metal. The higher the melting point is, the greater the attraction is. Moreover, we think that for a binary metal potential, if the attraction between the pure metal atoms is too large, there are deviations in the attraction between binary metal atoms. These deviations lead to anomalous results when the potential is used to carry out an MD simulation of the alloying of the binary metal system. For example, when the W/Cu EAM potential presented by Gong et al. [19] was used to carry out MD simulations of the melting behavior of W metal, the simulated melting point of W was 4625 K, which was much higher than the experimental melting point of W (3695 [21]). If this W/Cu EAM potential is used to simulate the direct alloying of W and Cu, diffusion between W and Cu can occur at room temperature [19], which is unrealistic. In contrast, the new W-Cu potential in this study can simulate the melting point of W at 3775 K, which is less than 80 K from the true melting point of W. This enables the new W-Cu potential to simulate the W-Cu alloying behavior, which agrees closely with our previous experimental results.

## 4. Conclusions

To study the diffusion mechanism for direct alloying between W and Cu in a temperature range close to the melting point of Cu and facilitate the preparation of W-Cu composite materials, a suitable W-Cu potential is constructed in this paper. This W-Cu potential takes the form of an F-S potential that combines the original F-S potential form for the W-W potential with the extended F-S potential form for the Cu-Cu potential and the W-Cu component. The construction process shows that to successfully develop an interatomic potential for an immiscible system, whether the new interatomic potential can accurately simulate the melting point of a high-melting-point constituent element in the system is a very important standard.

Validation by theoretical calculations and experiments shows that the new W-Cu potential is reliable and the method of developing the W-Cu potential is reasonable. First, the new W-Cu potential can be used to accurately simulate physical properties, such as the cohesive energies, lattice constants, vacancy formation energies, elastic constants and melting points, of pure W and Cu through the molecular dynamics method (MD). The calculated melting points of 1325 K for Cu and 3775 K for W, are relatively consistent with the actual melting points of Cu (1358 K) and W (3695 K), respectively. Second, the new W-Cu potential can be successfully used to simulate direct alloying of W and Cu through MD. The simulation results show that the Cu atoms mainly diffuse into the W metal rather than the W atoms diffusing into the Cu metal during alloying, and a disordered structure develops in the Cu metal and makes the Cu atoms break free from binding of the metal, promoting diffusion. These simulation results are in good agreement with experimental results.

## Figures and Tables

**Table 1:** Obtained parameters for the Cu-Cu, W-W and W–Cu components of the W-Cu potential.

	Cu-Cu	σ	W-W	σ	W-Cu	σ
*A*(eV·Å^−1^)	0.732311	0.017181	1.830783	0.009250	−0.851995	0.043304
*d*(Å)	3.983654	0.036376	4.414750	0.008165	4.270928	0.069296
*c*(Å)	3.777141	0.010236	3.252641	0.003195	3.761555	0.073371
*c_0_*(eV·Å^−2^)	2.126664	0.063405	55.424876	3.755775	20.500143	1.524143
*c_1_*(eV·Å^−3^)	−0.980911	0.049285	−40.097017	4.545578	−26.984701	1.276844
*c_2_*(eV·Å^−4^)	0.002647	0.000674	7.453343	1.459553	11.890381	0.069772
*c_3_*(eV·Å^−5^)	0.012049	0.004721	0	0	−1.744063	0.02767
*c_4_*(eV·Å^−6^)	0.006451	0.001685	0	0	0	0
*b*(Å^−2^)	0.525082	0.029941	0	0	0	0

**Table 2:** Comparison between the bulk and physical properties of pure Cu and W calculated using the present potentials and their reference experimental values.

	Cu		W	
	Experimental	Calculated	Experimental	Calculated
*a* (Å)	3.615	3.615	3.16475	3.165
*Ec* (eV)	3.54	3.540	8.66	8.660
*C*_11_ (Mbar)	1.7	1.700	5.326	5.326
*C*_12_ (Mbar)	1.225	1.227	2.05	2.050
*C*_44_ (Mbar)	0.758	0.759	1.631	1.631
*E_v_* (eV)	1.28	1.294	3.95	3.551

**Table 3:** Lattice constant *a* (Å) and cohesive energy *Ec* (eV) of two fictitious solid solutions calculated through the MD method based on the new W-Cu potential and ab initio method.

System	Structure	ab Initio Calculation		Present Study	
		*a* (Å)	*Ec* (eV)	*a* (Å)	*Ec* (eV)
Cu3W	L1_2_	3.756	4.229	3.755	4.230
CuW	B2	3.030	5.421	3.247	5.510
CuW3	L12	3.910	7.041	4.132	7.213

## Data Availability

The raw/processed data required to reproduce these findings can be found in this paper.

## Supplementary Materials

The following are available online at https://www.mdpi.com/article/10.3390/ma14205988/s1, Figure S1: The structures of the fictitious W-Cu solid solutions, Figure S2: Calculated total energy versus average atomic volume for the B2 and L12 structures, Figure S3: Pair correlation functions for (a) initial structure and (b) W-Cu interfacial structure obtained through alloying at 1200 K for 2 ns, Figure S4: Fine-scale density profiles (ρ (Z)) for W and Cu near and at the W-Cu interface constructed through direct alloying at different temperatures, Table S1: Parameters obtained for the W-W potential parts of the W-Cu potential, Table S2: Lattice constant a (Å) and cohesive energy Ec (eV) of two fictitious solid solutions calculated through the ab initio method.
