# Supplementary material for: Construction of an n-Body Potential for Revealing the Atomic Mechanism for Direct Alloying of Immiscible Tungsten and Copper
Source: Materials (Basel). 2021 Oct 12;14(20):5988. doi: 10.3390/ma14205988 (PMC8537307; doi:10.3390/ma14205988)
Supplement: Supplementary file 1 [file materials-14-05988-s001.zip › materials-1388663-supplementary.pdf]

Supporting Information for

Construction of an *n*-body potential for revealing the atomic mechanism for direct alloying of immiscible tungsten and copper

Tao Zeng, Fei Li and Yuan Huang*

Institute of Advanced Metallic Materials, School of Materials Science and Engineering, Tianjin University, Tianjin 300350, People's Republic of China

A. Calculation of physical properties using the new *n*-body potential

The relationships between the *n*-body potential and the physical properties, such as the cohesive energy, lattice constant, pressure, elastic constant, vacancy formation energy and bulk modulus, have been proposed by some researchers [S1, S2]. The calculation methods have been modified and are briefly described here. The cohesive energy (E_c) can be obtained by calculating the total energy after determining the lattice constant (a) of the structure in an equilibrium state, which can be expressed by Formulas (S1) and (S2).

$$\boldsymbol{E}_c = \frac{1}{2}\sum_{j\neq i} \boldsymbol{V}\left(\boldsymbol{r}_{ij}\right) + \boldsymbol{F}\left(\rho\right) \tag{S1}$$

$$\rho = \sum_{j\neq i} \phi\left(\boldsymbol{r}_{ij}\right) \tag{S2}$$

where $\boldsymbol{r}_{ij}$ refers to the distance between atom $\boldsymbol{i}$ and atom $\boldsymbol{j}$, and the pair-potential $\boldsymbol{V}\left(\boldsymbol{r}_{ij}\right)$, embedding energy $\boldsymbol{F}(\rho)$ and electron density $\phi(r_1)$ have the corresponding functional expressions for different potentials (see Formulas (2)-(11)). The pressure in the equilibrium state, which should equal zero, is being defined by:

$$\boldsymbol{P} = -\frac{d\boldsymbol{E}_C}{d\boldsymbol{\Omega}} \tag{S3}$$

where Ω represents the atomic volume, which is $\boldsymbol{a}^3/2$ for the bcc structure and $\boldsymbol{a}^3/4$ for the fcc structure. To characterize the resistance to volume change of the material,

the bulk modulus $\boldsymbol{B}$ can be defined as follows:

$$\boldsymbol{B} = -\Omega \frac{d\boldsymbol{P}}{d\Omega} \tag{S4}$$

The vacancy formation energy can be defined as the change in the energy caused by removing a random atom j from a perfect crystal structure and can be calculated by Formula (S5),

$$\boldsymbol{E}_{\mathrm{v}} = \sum_{i \neq j} \left\{ -\frac{1}{2} \boldsymbol{V}\left(\boldsymbol{r}_{ij}\right) + \boldsymbol{F}\left[\rho_{i0} - \phi\left(\boldsymbol{r}_{ij}\right)\right] - \boldsymbol{F}\left(\rho_{i0}\right) \right\} \tag{S5}$$

where ρ_{i0} refers to the total electron density at atom $\boldsymbol{i}$ in an equilibrium state, which is contributed by the surrounding atoms. The elastic constants of the equilibrium state are expressed as

$$\boldsymbol{C}_{\alpha\beta\upsilon\nu} = \frac{1}{\Omega} \sum_{i=1}^{n} \left[\boldsymbol{B}^{i}_{\alpha\beta\upsilon\nu} + \boldsymbol{F}'\left(\rho_{i0}\right) \boldsymbol{W}^{i}_{\alpha\beta\upsilon\nu} + \boldsymbol{F}''\left(\rho_{i0}\right) \boldsymbol{D}^{i}_{\alpha\beta} \boldsymbol{D}^{i}_{\upsilon\nu} \right] \tag{S6}$$

where $\boldsymbol{n}$ is the number of atoms in the crystal cell. The $\mathrm{F}'\left(\rho_{i0}\right) = \frac{dF\left(\rho_{i0}\right)}{d\rho_{i0}}$, $\mathrm{F}''\left(\rho_{i0}\right) = \frac{d^2F\left(\rho_{i0}\right)}{\left(d\rho_{i0}\right)^2}$ and ρ_{i0} terms in Formula (S6) are the total electron density at atom $\boldsymbol{i}$ in the equilibrium state. The $B^{i}_{\alpha\beta\upsilon\nu}$, $W^{i}_{\alpha\beta\upsilon\nu}$, $D^{j}_{\alpha\beta}$ and $D^{j}_{\upsilon\nu}$ terms in Formula (S6) are given by Formulas (S7), (S8) and (S9), respectively,

$$\boldsymbol{B}^{i}_{\alpha\beta\upsilon\nu} = \frac{1}{2} \sum_{j \neq i} \left\{ \left[\frac{d^2\boldsymbol{V}\left(\boldsymbol{r}_{ij}\right)}{d\boldsymbol{r}_{ij}^2} - \frac{d\boldsymbol{V}\left(\boldsymbol{r}_{ij}\right)}{d\boldsymbol{r}_{ij}} \frac{1}{\boldsymbol{r}_{ij}} \right] \frac{\boldsymbol{r}^{\alpha}_{ij} \boldsymbol{r}^{\beta}_{ij} \boldsymbol{r}^{\upsilon}_{ij} \boldsymbol{r}^{\nu}_{ij}}{\boldsymbol{r}^{2}_{ij}} \right\} \tag{S7}$$

$$\boldsymbol{W}^{i}_{\alpha\beta\upsilon\nu} = \frac{1}{2} \sum_{j \neq i} \left\{ \left[\frac{d^2\phi\left(\boldsymbol{r}_{ij}\right)}{d\boldsymbol{r}_{ij}^2} - \frac{d\phi\left(\boldsymbol{r}_{ij}\right)}{d\boldsymbol{r}_{ij}} \frac{1}{\boldsymbol{r}_{ij}} \right] \frac{\boldsymbol{r}^{\alpha}_{ij} \boldsymbol{r}^{\beta}_{ij} \boldsymbol{r}^{\upsilon}_{ij} \boldsymbol{r}^{\nu}_{ij}}{\boldsymbol{r}^{2}_{ij}} \right\} \tag{S8}$$

$$\boldsymbol{D}^{i}_{\alpha\beta} = \sum_{j \neq i} \frac{d\phi\left(\boldsymbol{r}_{lj}\right)}{d\boldsymbol{r}_{lj}} \frac{\boldsymbol{r}^{\alpha}_{lj} \boldsymbol{r}^{\beta}_{lj}}{\boldsymbol{r}_{lj}} \tag{S9}$$

where the superscripts α, β, υ and ν =1, 2 and 3 are the notation of the fourth-order tensor and stand for the x-, y- and z-axes, respectively. In addition, the Cauchy pressure $\boldsymbol{P}_C$ is defined as:

$$\boldsymbol{P}_C = \frac{1}{2}(\boldsymbol{C}_{1122} - \boldsymbol{C}_{2323}) \tag{S10}$$

With the experimental values of these physical properties, the process for determining the parameters for the W-W potential part of the W-Cu potential is as follows. First, with the effective initial values of *c* and *d* between the second- and third-nearest neighbors of the structure of W, the values of the parameters A, c_0, c_1 and c_2 can be obtained by solving a set of linear equations, which consist of Formulas (S1, S3, S4 and S10). Second, the value of *d* can be determined by iterating Formula (S6) with the experimental value of $\boldsymbol{C}_{2323}$. Then, the value of *c* can be determined by Formula (S5) with the range $0.3\boldsymbol{E}_c \le \boldsymbol{E}_\text{v} \le 0.5\boldsymbol{E}_c$ proposed by Finnis et al. [S2]. The results for the parameters of the W-W potential parts of the W-Cu potential are listed in Table S1.

Table S1: Parameters obtained for the W-W potential parts of the W-Cu potential.

	W-W
A(eV·Å^{-1})	1.830783
d(Å)	4.41
c(Å)	3.25
c_0(eV·Å^{-2})	55.424876
c_1(eV·Å^{-3})	-40.097017
c_2(eV·Å^{-4})	7.453343
c_3(eV·Å^{-5})	0
c_4(eV·Å^{-6})	0
b(Å^{-2})	0

B. The fictitious W-Cu solid solution structures and corresponding calculation

process

Fig. S1 shows the constructed B2 and $L1_2$ structures of the fictitious W-Cu solid solutions. First-principles calculations were carried out by using the well-established Vienna ab initio simulation package (VASP), the projector-augmented wave pseudopotentials were employed to describe the interaction between ions and electrons, and the generalized gradient approximation (GGA) [S3] proposed by Perdew and Wang was used to describe the exchange and correlation items. The integration in the Brillouin zone used the Monkhorst-Pack scheme, and the *k* lattice point of invert space was selected as 11*11*11. The cutoff energies for both B2 and $L1_2$ structures were set to 500 eV. Then, the correlations between the total energy and average atomic volume for B2 and $L1_2$ structures were calculated and are shown in Fig. S2. The cohesive energies and lattice constants of the B2 and $L1_2$ structures can be obtained from the lowest points of the corresponding curves in Fig. S2 and are shown in Table S2 after correction [S4].

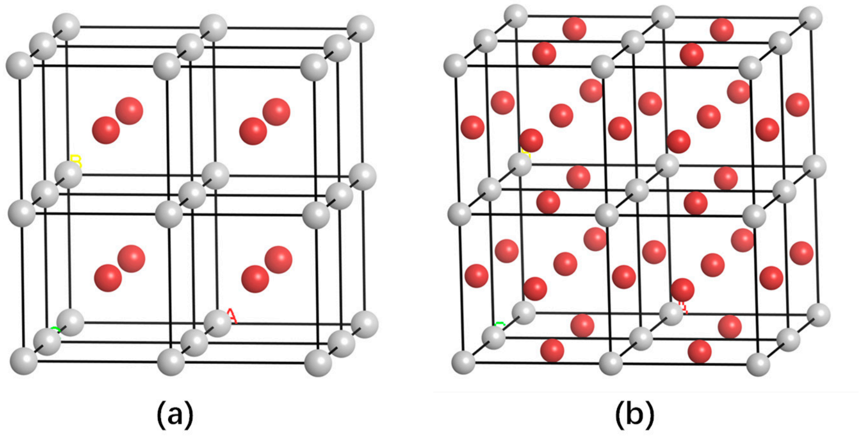

Fig. S1. The structures of the fictitious W-Cu solid solutions: (a) B2 and (b) $L1_2$. The red and gray atoms represent Cu and W atoms, respectively.

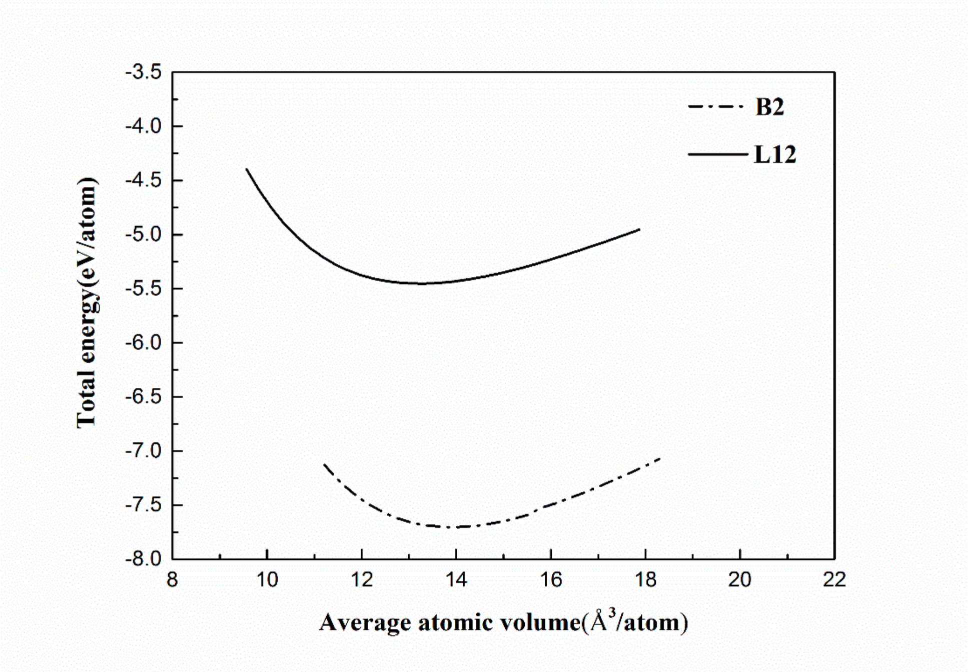

Fig. S2. Calculated total energy versus average atomic volume for the B2 and $L1_2$ structures.

Table S2: Lattice constant *a* (Å) and cohesive energy *Ec* (eV) of two fictitious solid solutions calculated through the ab initio method.

system	structure	*a*(Å)	*Ec*(eV)
Cu3W	$L1_2$	3.756	4.229
CuW	B2	3.030	5.421

C. Analysis of the pair correlation function of the W/Cu interfacial structure at different times

To determine the degree of disorder of atoms in the layer between W and Cu, the pair correlation functions $g(r)$ were calculated by Formula (S11) [S5] according to the MD simulation results when the distance from one atom was *r*. The pair correlation

functions $g(r)$ can reflect the probability of finding another atom around one atom in a structure.

$$g(r) \approx \frac{1}{\rho_0} \frac{n_{(r)}}{4\pi r^2 \delta r} \tag{S11}$$

where ρ_0 is the atomic density of the ideal crystal and $n(r)$ is the number of particles in a spherical shell with radius *r* to $r + \delta r$. The calculated pairwise correlation functions *g*(***r***) for the initial crystal structure at 300K and the W/Cu interfacial structure upon annealing at 1200 K for 2ns are shown in Fig. S3. Fig. S3 shows that, compared with the initial crystal structure, the sharp peaks of Cu disappear after 2 ns of alloying (see the red curves in Fig. S3), which means that the structure of Cu is in a state of disorder at the alloying temperature. At this time, Cu has higher energy and a looser structure [S6], which makes it easier for the Cu atoms to break away from the binding of the Cu matrix and enter W. In addition, Fig. S3 shows that the W–Cu partial *g*(*r*) appears (see the blue curves in Fig. S3), which proves the occurrence of diffusion between W and Cu and that the diffusion structure between W and Cu exists in an amorphous form during alloying. Last, Fig. S3 shows that the structure of W at the interface has also become disordered (see the black curves in Fig. S3), which should be caused by the penetration of Cu into W.

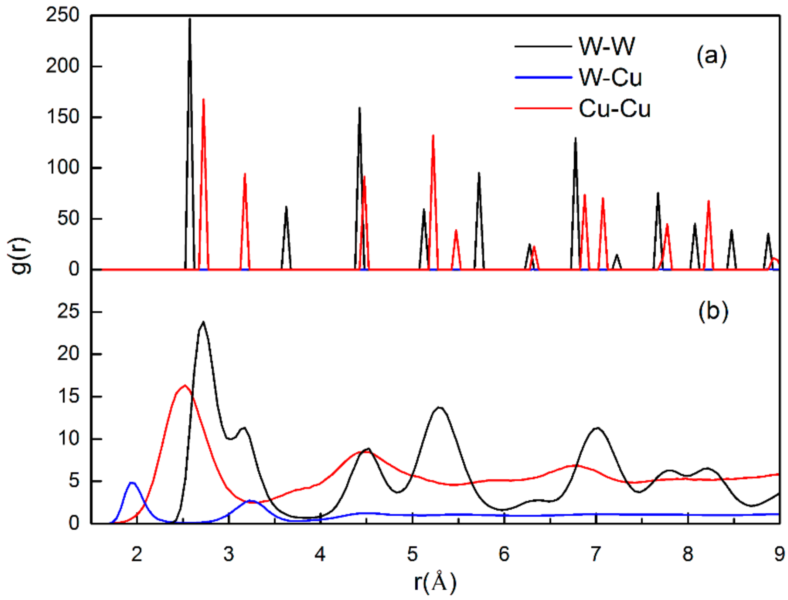

Fig. S3. Pair correlation functions for (a) initial structure and (b) W–Cu interfacial structure obtained through alloying at 1200 K for 2 ns, where the red curves represent the Cu-Cu partial *g*(*r*), the black curves represent the W-W partial *g*(*r*), and the blue curve represents the W–Cu partial *g*(*r*).

D. Analysis of the fine-scale density profiles for the W-Cu interface during direct alloying at different temperatures.

The fine-scale density profiles ($\rho_\alpha(z)$) characterize the distribution of atomic number density along the direction of diffusion [S7], which can be used to analyze the microstructure characteristics of the diffusion interfacial region between W and Cu. The corresponding calculation formula can be expressed by:

$$\rho_\alpha(Z) = \frac{\langle N_Z^\alpha \rangle}{A\Delta Z} \quad \text{(S12)}$$

where $\langle N_Z^\alpha \rangle$ is the average number of α atoms in the particular bin from $(z - \Delta z/2)$ to $(z + \Delta z/2)$, Δz is the distance between adjacent bins, and A represents the interfacial

area. For a crystal structure, the curve of the fine-scale density profile oscillates periodically, and for a disordered structure, the curve becomes smoother. According to Formula (S12), $\rho_\alpha(\boldsymbol{Z})$ after alloying W and Cu is calculated based on the MD simulation results. Fig. S4 shows the obtained curves of $\rho_\alpha(\boldsymbol{Z})$ for W and Cu during direct alloying at and near the interface at temperatures of 900 K, 1000 K, 1100 K, 1200 K and 1300 K.

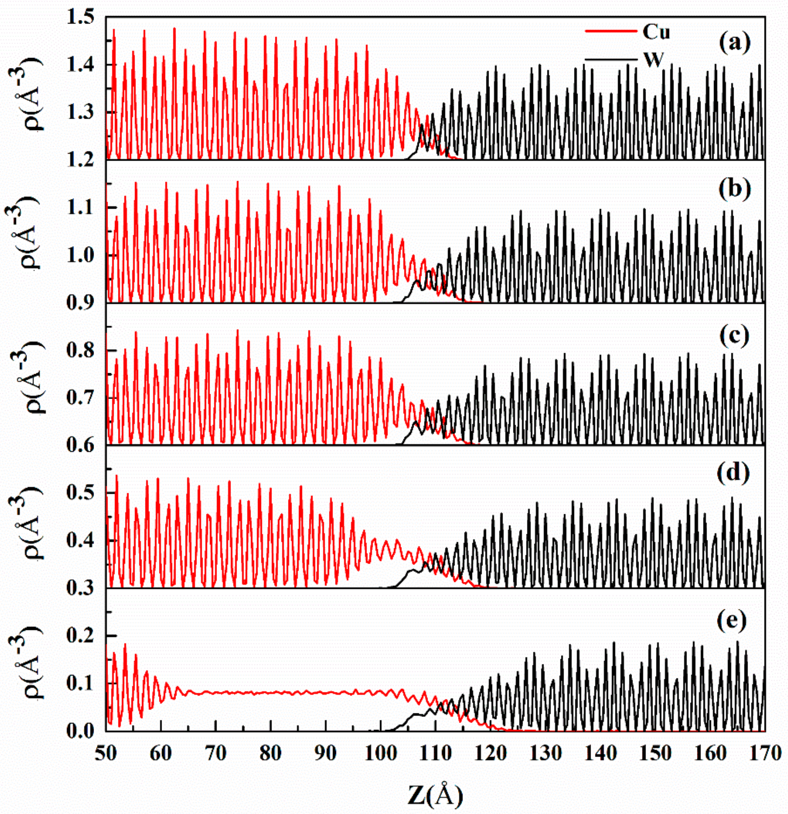

Fig. S4. Fine-scale density profiles (ρ (Z)) for W and Cu near and at the W-Cu interface constructed through direct alloying at different temperatures: (a) 900 K, (b) 1000 K, (c) 1100 K, (d) 1200 K, and (e) 1300 K. The red curves represent Cu, and the black curves

represent W.

Fig. S4 shows that with increasing alloying temperature, the periodicity of the $\rho_\alpha(\boldsymbol{Z})$ curve of Cu decreases gradually and even disappears at 1300 K (see Fig. S4(e)). However, except for a weakening at the interface, the periodicity of the $\rho_\alpha(\boldsymbol{Z})$ curve of W is maintained.

E. The nomenclature list for all acronyms and parameters.

Table S3: The nomenclature list for all acronyms.

Acronyms	Meaning
MD	molecular dynamics
PFMs	plasma facing materials
HTSIA	high-temperature structure induced alloying
EAM	embedded atom method
fcc	face-centered cubic
bcc	body-centered cubic
VASP	Vienna ab initio simulation package
TPS	two-phase simulation
OPS	one-phase simulation
NPT	isothermal-isobaric
LAMMPS	large-scale atomic molecular massively parallel simulator
HAADF-STEM	high-angle annular dark-field scanning transmission electron microscopy
FIB	focused ion beam
XRD	X-ray diffraction
SAED	selected areas of electron diffraction

Table S4: The nomenclature list for all parameters.

Parameters	Meaning
ΔHf	heat of formation
T_{mCu}	melting point of Cu
E_{tot}	total energy of the system
$V_{ij}(r_{ij})$	conventional central pair potential
r_{ij}	distance between atom *i* and *j*
$F_i(\rho_i)$	embedded energy
ρ_i	electronic density at atom *i*
c	cutoff distance of the pair potential

d	electronic density function
a	lattice constant
E_C	cohesive energy
E_v	vacancy formation energy
B	bulk modules
C_{11}, C_{12}, and C_{44}	elastic constants
P	pressure
B2, $L1_2$	Strukturbericht Designation
$g(r)$	pair correlation function
$\rho_\alpha(z)$	fine-scale density profile

References

[S1] J.H. Li, X.D. Dai, S.H. Liang, K.P. Tai, Y. Kong, B.X. Liu, Interatomic potentials of the binary transition metal systems and some applications in materials physics, Phys. Rep., 455 (2008) 1-134.

[S2] M.W. Finnis, J.E. Sinclair, A simple empirical N-body potential for transition metals, Philos. Mag. A, 50 (1984) 45-55.

[S3] J.P. Perdew, Y. Wang, Accurate and simple analytic representation of the electron-gas correlation energy, Phys. Rev. B, 45 (1992) 13244-13249.

[S4] R.F. Zhang, L.T. Kong, H.R. Gong, B.X. Liu, Comparative study of metastable phase formation in the immiscible Cu–W system byab initiocalculation andn-body potential, J. Phys.: Condens. Matter, 16 (2004) 5251-5258.

[S5] Y. Waseda, The Structure of Non Crystalline Materials: Liquids and Amorphous Solids, New York, 1980.

[S6] D.F. Jiang, J.Y. Long, M.Y. Cai, Y. Lin, P.X. Fan, H.J. Zhang, M.L. Zhong, Femtosecond laser fabricated micro/nano interface structures toward enhanced bonding strength and heat transfer capability of W/Cu joining, Mater. Des., 114 (2017) 185-193.

[S7] X. Gan, S. Xiao, H. Deng, X. Sun, X. Li, W. Hu, Atomistic simulations of the Fe(001)–Li solid–liquid interface, Fusion Eng. Des., 89 (2014) 2894-2901.
